# Frequency-specific stimulations induce reconsolidation of long-term potentiation in freely moving rats

**DOI:** 10.1186/s13041-016-0216-4

**Published:** 2016-03-25

**Authors:** Reiko Okubo-Suzuki, Yoshito Saitoh, Mohammad Shehata, Qi Zhao, Hiroshi Enomoto, Kaoru Inokuchi

**Affiliations:** Department of Biochemistry, Faculty of Medicine, Graduate School of Medicine & Pharmaceutical Sciences, University of Toyama, 2630 Sugitani, Toyama, 930-0194 Japan; Japan Science and Technology Agency, CREST, University of Toyama, Toyama, 930-0194 Japan

**Keywords:** Reconsolidation, Long-term potentiation, Theta stimulation, NMDA receptors

## Abstract

**Background:**

When consolidated memories are retrieved, they become labile and a new protein synthesis-dependent reconsolidation process is required to restabilize these memories. So far, most studies conducted on reconsolidation rely on the analyses of animal behavior, leaving the synaptic mechanisms that underlie reconsolidation largely unclear. Here, we examined whether the reconsolidation process occurs in hippocampal long term potentiation (LTP), as a synaptic model that is correlated with long term memories (LTM).

**Results:**

We employed LTP system in the dentate gyrus of freely moving rats that lasts for weeks simulating LTM. LTP was induced by high frequency stimulation at 400 Hz (HFS400), and as a reactivation stimulation, we tested a low frequency stimulation at 0.1 Hz (LFS0.1), a theta stimulation at 8 Hz (TS8), or HFS400. Unlike HFS400 reactivation, both LFS0.1 and TS8 induced a reconsolidation-like phenomenon and rendered the LTP labile to protein synthesis inhibition by anisomycin. Without reactivation, LTP remained unaffected by protein synthesis inhibition. In addition, the TS8-induced LTP reconsolidation was NMDAR dependent.

**Conclusion:**

Our results indicate that, as with behavioral LTM, there are boundary conditions for LTP reconsolidation where only a certain range of frequency stimulations as reactivation can destabilize the consolidated LTP. This LTP reconsolidation system will be useful for future elucidation of the synaptic reconsolidation mechanism.

## Background

When a consolidated memory is retrieved in proper conditions, the memory becomes labile or destabilized, and a protein synthesis is required for restabilization of that memory; a process referred to as “reconsolidation” [1]. The reconsolidation process is observed in a variety of animal species and learning tasks, therefore, it is considered as a fundamental memory process [2, 3]. Interference with the restabilization of the memory leads to retrograde amnesia and hence, might be the basis for the treatment of certain psychiatric disorders, such as post-traumatic stress disorder and drug addiction [4]. However, when proper conditions are not met, memory retrieval does not induce memory destabilization and hence, interference will have no amnesic effect, as the memory is still intact in the first place. These conditions are referred to as the “boundary conditions” only beyond which reconsolidation can occur [3]. Most of the previous reports studied the boundary conditions and the nature of memory destabilization were performed through behavioral analysis. However, the cellular and the synaptic nature of memory destabilization and the boundary conditions are greatly unclear and needs profound elucidation.

The synaptic phenomena, long-term potentiation (LTP) and long-term depression (LTD), are correlated with memory encoding [5]. In parallel with the distinction of short-term memory (STM) and long-term memory (LTM), LTP is also divided into a transient early phase LTP (E-LTP) and a stabilized, RNA- and protein synthesis-dependent late phase LTP (L-LTP) [6]. Moreover, LTD and LTP can inactivate and reactivate an artificial memory, respectively, proofing a causal link between these synaptic processes and memory [7]. Consequently, we asked whether the reconsolidation process also occurs in hippocampal LTP and are there boundary conditions for its occurrence? In other words, does electric stimulation after LTP induction work as reactivation (corresponding to memory retrieval) returning the LTP to a labile state, and hence, a new protein synthesis process is required for LTP restabilization? Also, is any electric stimulation can induce such proposed LTP reconsolidation?

The aim of this study is to answer these questions using an in vivo LTP in the hippocampal dentate gyrus of freely moving rats. In contrast to other LTP systems, this system is unique as it allows us to observe changes in the population spike amplitude of the field excitatory postsynaptic potential (fEPSP) at a time course of days to weeks similar to behavioral experiments [8]. We will show that low frequency stimulation (LFS) and theta stimulation (TS), but not high frequency stimulation (HFS) have ability to induce a reconsolidation-like phenomenon in our LTP system.

## Results

### Optimization of protein synthesis inhibition

**Fig. 1:**
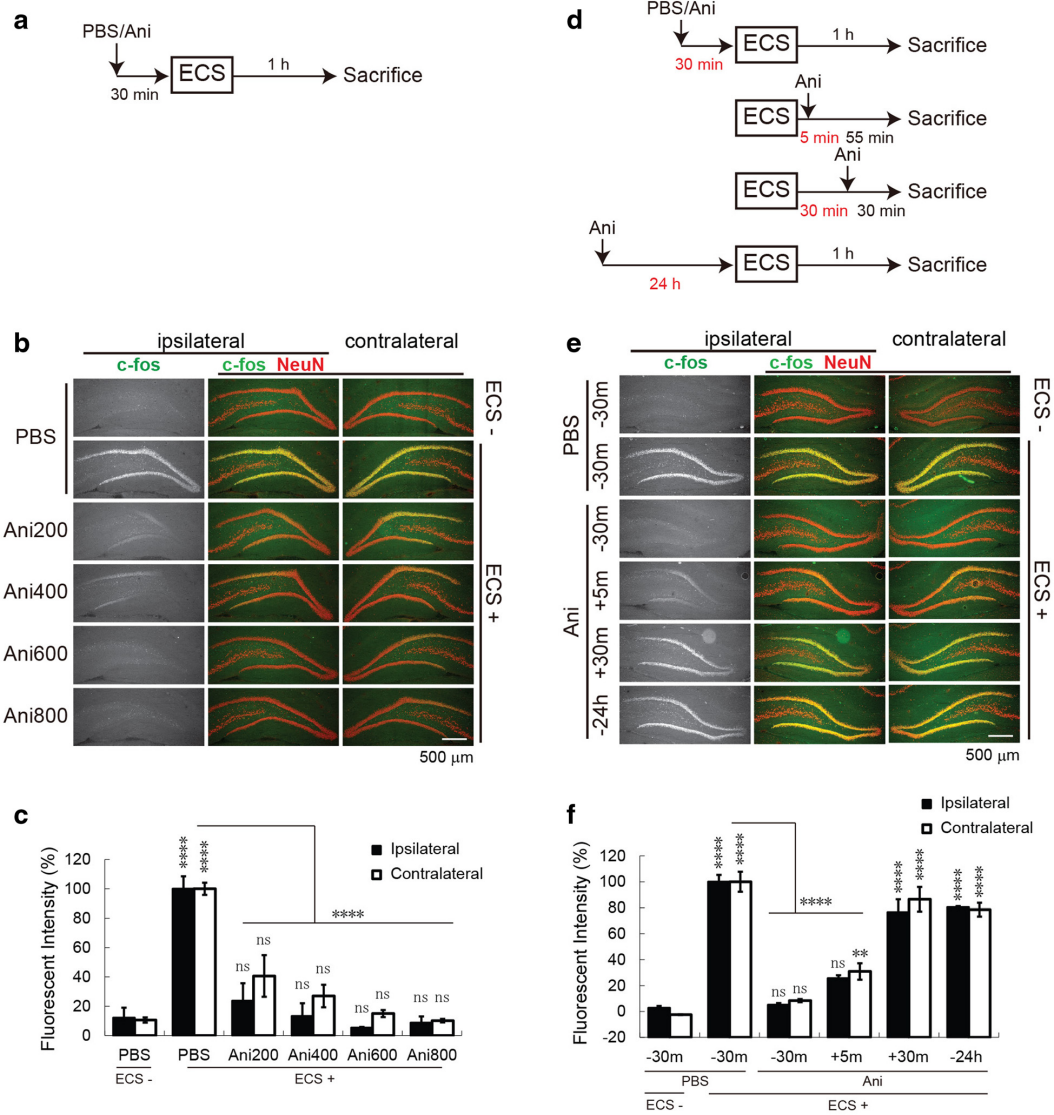
Optimization of protein synthesis inhibition. **a**–**c** The effect of anisomycin (Ani) dose on protein synthesis inhibition following electric convulsive stimulation (ECS). **d**–**f**, The effect of 600 μg anisomycin injection time on protein synthesis inhibition following ECS. **a** and **d** Experimental design. **b** and **e** Representative immunohistochemically stained images of c-fos (*green*) and NeuN (*red*) expression in the dentate gyri ipsi- and contra-lateral to the i.c.v. injection side. **c** and **f**, Quantitation of c-fos signal in (**b**) and (**e**), respectively. *n* = 3–4 rats per condition. (** *p* < 0.01; **** *p* < 0.0001; ns, not significant (*p* > 0.05); two-way ANOVA, Tukey’s posthoc for within group test compared to PBS group without ECS except otherwise indicated and Sidak posthoc for test between ipsi- and contra-lateral sides). No significant difference between ipsi- and contra-lateral sides at all points. *Error bars* indicate SEM

As a dentate gyrus (DG) LTP in freely moving rats was employed in this study [8–11], the pharmacological drugs were injected into the lateral ventricle (i.c.v.) not to interfere with fEPSP recording. First, we investigated the proper dose of the protein synthesis inhibitor, anisomycin, delivered into the lateral ventricle that can diffuse and inhibit protein synthesis in the dorsal hippocampus. Protein synthesis was assessed using the electroconvulsive stimulation (ECS)-induced c-fos, an immediate early gene, expression in the DG neurons, labeled by NeuN. Anisomycin, injected 30 min before ECS, showed a dose-dependent decrease in c-fos expression compared to phosphate buffered saline (PBS), an hour after ECS (Fig. 1 a and b). The decrease in c-fos expression reached a plateau at 600 μg anisomycin (reaching about 95 % inhibition in the ipsilateral side where recording was done) (two-way ANOVA: treatment F_(5, 26)_ = 54.98, *P* < 0.0001) (Fig. 1c). Next, we tested the effect of 600 μg anisomycin injection time on ECS-induced c-fos expression (Fig. 1d). A significant decrease in c-fos expression compared to PBS was obtained when anisomycin was injected 5 min, but not 30 min, after ECS (two-way ANOVA: treatment timing F_(5, 24)_ = 124.1, *P* < 0.0001) (Fig. 1e and f). From the above, a dose of 600 μg anisomycin injected 5 min after electric stimulation was used in the following experiments. In addition, anisomycin injected 24 h before ECS did not show a significant decrease in the ECS-induced c-fos expression compared to PBS (Fig. 1d and f). This indicates that the anisomycin effect is only transient and has no long lasting effect.

**Fig. 2:**
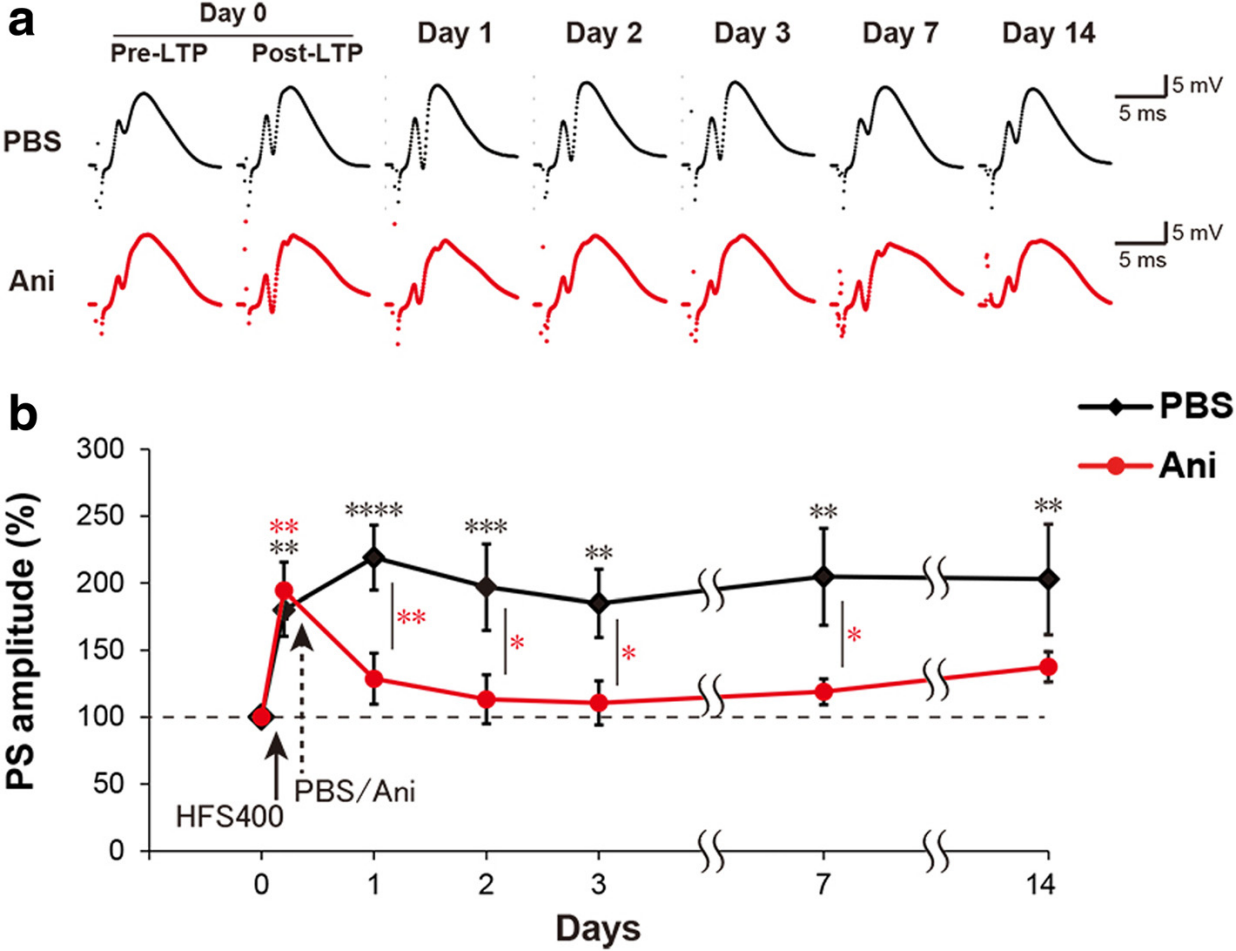
Anisomycin blocks the PS-LTP consolidation in freely moving rats. **a** Trace samples of recorded fEPSP signals. **b** The average of the population spike (PS) amplitude recorded at each time point. Anisomycin (Ani) or PBS (Vehicle) was injected 5 min after HFS400. *n* = 8–9 per group. RM two-way ANOVA, Newman-Keuls posthoc for within group test compared to pre-LTP and Fisher’s LSD for between PBS and Ani groups test (** *p* < 0.01; *** *p* < 0.001; **** *p* < 0.0001). *Error bars* indicate SEM

Next, we tested whether i.c.v. injection of 600 μg anisomycin, 5 min after LTP induction, can inhibit protein synthesis during the LTP consolidation and hence, block L-LTP maintenance, as previously report from direct injection [12]. LTP was induced by high frequency stimulation at 400 Hz (HFS400) delivered through the stimulating electrode implanted in the entorhinal cortex [8]. HFS400 resulted in a long lasting potentiation, maintained for at least 2 weeks, in the population spike (PS) amplitude (PS-LTP) of the evoked fEPSP recorded from the electrode implanted in hippocampal DG (Repeated measures (RM) two-way ANOVA: time F_(6,90)_ = 5.231, *P* = 0.0001) (Fig. 2a and b). When anisomycin was injected after LTP induction, the PS-LTP significantly decreased compared to PBS and to almost baseline level (RM two-way ANOVA: interaction F_(6,90)_ = 3.136, *P* = 0.0077). The anisomycin effect was observed one day after LTP induction and PS amplitude was not significantly above baseline for two weeks (Fig. 2a and b). Therefore, these conditions for protein synthesis inhibition were sufficient to completely block LTP maintenance in consistence with the literature.

### Low frequency stimulation induces PS-LTP reconsolidation

**Fig. 3:**
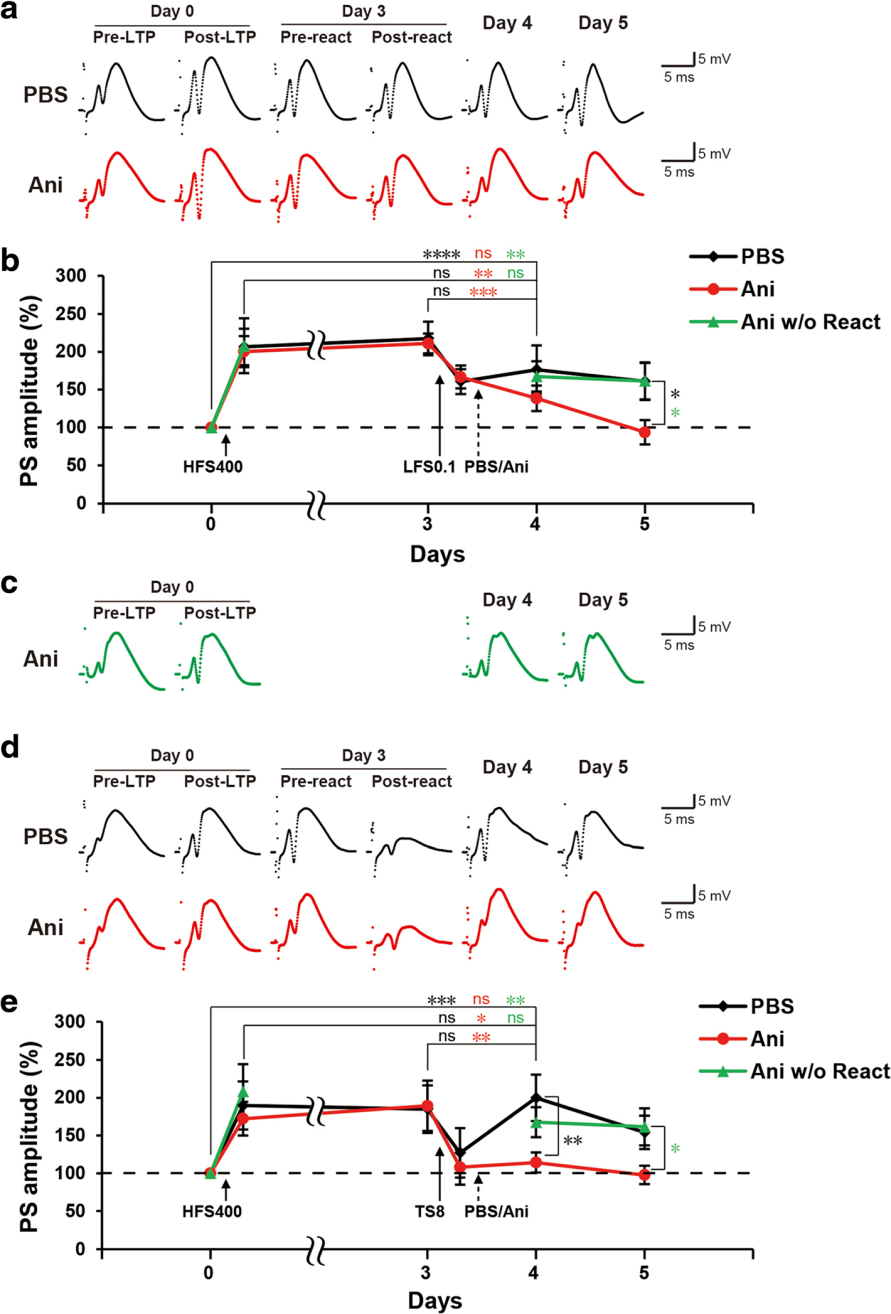
Low frequency and theta stimulations induce PS-LTP reconsolidation. **a**-**b** Low frequency-induced PS-LTP reconsolidation. **d**-**e** Theta stimulation-induced PS-LTP reconsolidation. Trace samples of recorded fEPSP signals recorded during the reactivation experiments with LFS0.1 (**a**), TS8 (**d**), or no reactivation (**c**). The average of the population spike (PS) amplitude recorded at each time point before and after LFS0.1 (**b**), TS8 (**e**), or no reactivation (**b** and **e**). Anisomycin (Ani) or PBS (Vehicle) was injected 5 min after reactivation or Ani injected without reactivation (Ani w/o React). *n* = 10–12 per group. RM two-way ANOVA, Newman-Keuls posthoc for within group test and Fisher’s LSD for between groups test (*p < 0.05; ** *p* < 0.01; *** *p* < 0.001; **** *p* < 0.0001; ns, not significant (*p* > 0.05)). *Error bars* indicate SEM

To establish a reconsolidation-like phenomenon in the in vivo PS-LTP model, we searched a stimulation protocols that destabilize a consolidated L-LTP and render it labile to anisomycin inhibition, to simulate the memory reactivation session performed in the behavioral reconsolidation experiments [1, 13]. We tested three previously reported protocols ranging from low to high frequency stimulations. First, we tested low frequency stimulation at 0.1 Hz (LFS0.1) that is previously reported to show a reconsolidation-like phenomenon in slice LTP model, where it resensitized LTP to protein synthesis inhibition [14]. As shown in the PBS group, the PS amplitude 1 day after LFS0.1 was not significantly different from the post-LTP or the pre-reactivation values and was significantly higher than the baseline level (RM two-way ANOVA: time F_(7,147)_ = 23.54, *P* < 0.0001) (Fig. 3a and b). Therefore, LFS0.1 per se did not affect the L-LTP maintenance. In the anisomycin group, the PS amplitude 1 day after LFS0.1 was significantly lower than the post-LTP and the pre-reactivation values and was not significantly different from the baseline level (Fig. 3a and b). The PS amplitude of the anisomycin group was significantly lower than the PBS group 2 days after LFS0.1 (RM two-way ANOVA: interaction F_(7,147)_ = 2.68, *P* = 0.0122) (Fig. 3a and b). When anisomycin was injected without reactivation session, the anisomycin effect was abolished and the PS amplitude was not significantly different from the post-LTP value and was significantly higher than the baseline level (RM two-way ANOVA: time F_(4,120)_ = 23.82, *P* < 0.0001) (Fig. 3b and c). The PS amplitude of the anisomycin group without reactivation was significantly higher than the anisomycin group 2 days after LFS0.1 (Fig. 3a, b and c). These data indicates that in agreement with the data from slice LTP model, LFS0.1 destabilizes L-LTP and hence, resensitizes it to protein synthesis inhibition.

### Theta stimulation induces PS-LTP reconsolidation

Next, we tested theta stimulation at 8 Hz (TS8) to simulate the theta rhythm reported to be critical for temporal coding/decoding of active neuronal ensembles and the modification of synaptic weights [15]. As shown in the PBS group, TS8 did not show any effect on L-LTP where the PS amplitude 1 day after TS8 was not significantly different from the post-LTP or the pre-reactivation values and was significantly higher than the baseline level (RM two-way ANOVA: time F_(6,126)_ = 9.822, *P* < 0.0001) (Fig. 3d and e). Interestingly, the post-reactivation recording after TS8 showed a consistent unusual change in the waveform that was only transient as the waveform returned to the normal shape one day later and in all other time points (Fig. 3d). In the anisomycin group, the PS amplitude after 1 day TS8 was significantly lower than the post-LTP and the pre-reactivation values and it was not significantly higher from the baseline level (Fig. 3d and e). The PS amplitude of the anisomycin group was significantly lower than the PBS group 1 day after TS8 (RM two-way ANOVA: interaction F_(6,126)_ = 2.646, *P* = 0.0189) (Fig 3d and e). When compared to the group injected with anisomycin without reactivation session, the PS amplitude of the anisomycin group 2 days after TS8 reactivation was significantly lower (Fig. 3c, d, and e). As with LFS0.1, these data indicates that theta stimulation also destabilizes L-LTP.

### High frequency stimulation does not induce PS-LTP reconsolidation

**Fig. 4:**
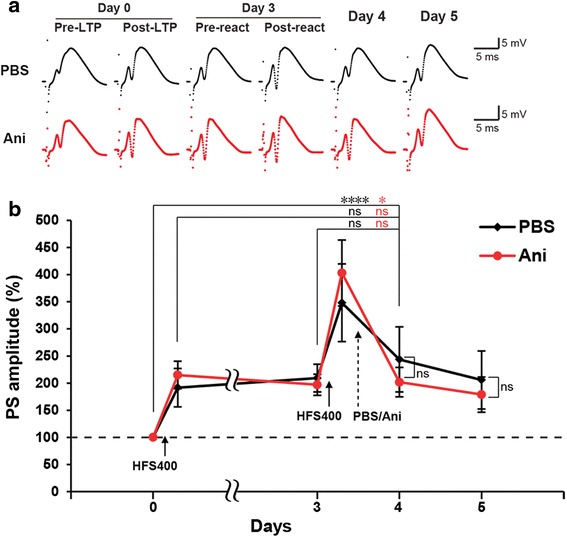
HFS does not induce PS-LTP reconsolidation. **a** Trace samples of recorded fEPSP signals recorded during the reactivation experiments with HFS400. **b** The average of the population spike (PS) amplitude recorded at each time point before and after HFS400 reactivation. Anisomycin (Ani) or PBS (Vehicle) was injected 5 min after reactivation. *n* = 10–12 per group. RM two-way ANOVA, Newman-Keuls posthoc for within group test and Fisher’s LSD for between groups test (*p < 0.05; **** *p* < 0.0001; ns, not significant (*p* > 0.05)). *Error bars* indicate SEM

Finally, we tested the HFS protocol at 400 Hz (HFS400), which was used to induce the L-LTP in the first place, and it induced long lasting LTP (Fig. 2). As shown in the PBS group, a second HFS400 did not show a considerable effect on L-LTP, and the PS amplitude 1 day after HFS400 reactivation was significantly higher than the baseline level (RM two-way ANOVA: time F_(6,120)_ = 23.54, *P* < 0.0001) (Fig. 4a and b). The post-reactivation recording after the HFS400 reactivation showed a significant increase in the PS amplitude compared to the pre-reactivation recording, however, this effect was only transient and the PS amplitude 1 day after the HFS400 reactivation was not significantly higher than the post-LTP or the pre-reactivation levels (Fig. 4a and b). In the anisomycin group, the PS amplitude 1 day after the HFS400 reactivation was not significantly different from the post-LTP, the pre-reactivation or the PBS group levels (RM two-way ANOVA: interaction F_(6,20)_ = 1.145, *P* = 0.3404), and it was significantly higher than the baseline level (Fig. 4a and b). Therefore, HFS did not re-sensitizing L-LTP to protein synthesis inhibition in contrast to LFS0.1 or TS8. These data indicates that electric stimulation per se is not sufficient to destabilize L-LTP and only a certain range of electric stimulations, at least within the range of 0.1 to 8 Hz, is necessary to render the formed L-LTP labile and sensitive to protein synthesis inhibition.

### The theta stimulation-induced PS-LTP reconsolidation is NMDA receptor dependent

**Fig. 5:**
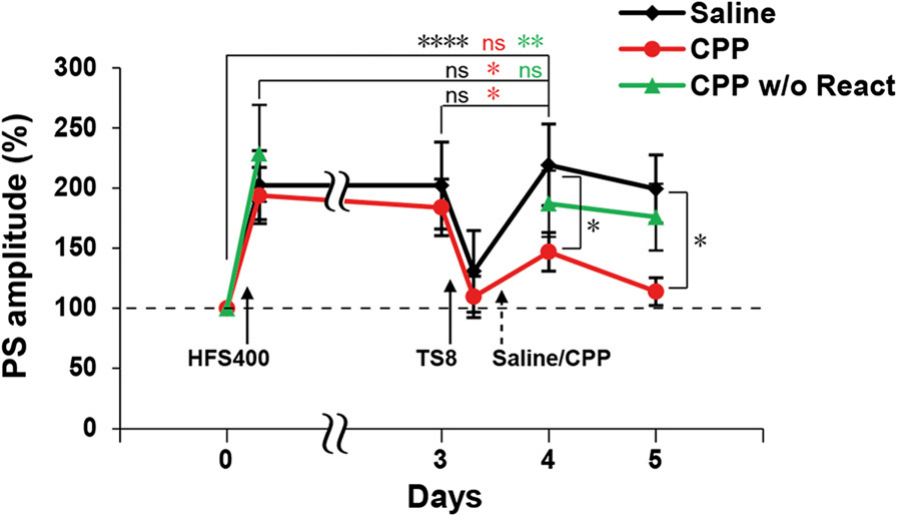
The theta stimulation-induced PS-LTP reconsolidation is NMDA receptor-dependent. The average of the population spike (PS) amplitude recorded at each time point before and after TS8 or no reactivation. CPP or Saline (Vehicle) was injected 5 min after reactivation or CPP injected without reactivation (React). *n* = 5–11 per group. RM two-way ANOVA, Newman-Keuls posthoc for within group test and Fisher’s LSD for between groups test (* *p* < 0.05; ** *p* < 0.01; **** *p* < 0.0001; ns, not significant (*p* > 0.05)). *Error bars* indicate SEM

In addition, we asked whether the PS-LTP reconsolidation and the behavioral reconsolidation share similar mechanisms. Reconsolidation on behavioral level is N-methyl-D-aspartate receptor (NMDAR) dependent. More specifically, GluN2B-containing and GluN2A-containing NMDARs are required in the memory destabilization and restabilization processes, respectively [16, 17]. Therefore, we examined the involvement of NMDARs in restabilization of the TS-induced PS-LTP reconsolidation model. Rats were i.c.v. injected with the NMDAR-antagonist, CPP, a highly potent NMDA antagonist showing selectivity for GluN2A-containing receptors, or saline 5 min after TS8 (Fig. 5). Unlike the saline group, the PS amplitude 1 day after TS8 of the CPP group significantly decreased compared to the post-LTP and the pre-reactivation levels, and it was not significantly higher from the baseline level (RM two-way ANOVA: interaction F_(6,114)_ = 3.551, *P* = 0.0029) (Fig. 5). The PS amplitude of the CPP group was significantly lower than the saline group 2 days after TS8 (Fig. 5). The CPP effect was observed only after reactivation, as CPP injected without reactivation session was not significantly different from the post-LTP or the saline group levels and it was significantly higher than the baseline (RM two-way ANOVA: interaction F_(4,92)_ = 26.3, *P* < 0.0001) (Fig. 5). These data indicates that activation of NMDARs is required for the restabilization of the PS-LTP after it has been destabilized with TS8 reactivation.

## Discussion

Synaptic potentiation in the amygdala after tone fear conditioning is reduced when reconsolidation is inhibited [18]. Also, in Aplysia, long-term facilitation of sensory-to-motor synapse is destabilized and restabilized after synaptic reactivation [19, 20]. This study extends these studies to LTP induced in the DG of mammalian hippocampus, showing that it can also undergo a reconsolidation process. Previous report, using CA1-LTP in hippocampal slice, showed that test pulse at 0.1 Hz reactivation of LTP render it sensitive to protein synthesis inhibitors [14]. The data from this report suggests the existence of reconsolidation-like phenomenon on the synaptic level. However, slice LTP can only be observed for several hours that limits the direct correlation with behavioral reconsolidation where LTM lasts for at least days. In contrast, the PS-LTP system in freely moving rats, which last for weeks, overcomes this shortcoming. Also, this PS-LTP system is associated with actin cytoskeletal reorganization characterized by an increase in F-actin content within dendritic spines and a path-specific alteration in postsynaptic actin-associated proteins; drebrin, and synaptopodin [8, 21]. These synaptic modifications were protein synthesis dependent and long-lasting for up to weeks [8].

Moreover, this study shows for the first time that there are boundary conditions for LTP reconsolidation. In behavioral reconsolidation experiments, several boundary conditions have been described that determine whether or not the reconsolidation process is elicited, including the memory age, the memory strength, and the reactivation strength [3]. Most relevant to this study is the reactivation strength, which is controlled in behavior by the length of the reactivation trial [13]. Although synaptic reactivation stimulations, used in this study to simulate memory retrieval, do not necessary reactivate memory in behaving animals, both synaptic and behavioral reconsolidation models may share the same molecular mechanisms. Indeed, memory destabilization, in successful reconsolidation behavioral paradigms, depends on protein degradation mechanisms including ubiquitin-proteasome system (UPS), and endocytosis [22, 23]. Meanwhile, according to the frequency of electric stimulation and the amount of calcium internalized, divergent signaling pathways can be elicited that finally favors a range of effects on the synapse from stabilization to destabilization [24]. HFS that is used to induce LTP, favors synapse stabilization through increasing local protein synthesis. In contrast, LFS that is usually used to induce LTD, favors synapse destabilization through increasing protein degradation, including UPS, endocytosis, and autophagy [24]. In this study, we showed that LFS and TS, but not HFS, induce LTP reconsolidation in an NMDA receptor-dependent manner. Therefore, the behavioral and the synaptic boundary conditions might converge at the molecular level.

## Conclusions

In this study, we showed that LTP induced in the DG of mammalian hippocampus undergoes a reconsolidation process, in a time course that matches behavioral reconsolidation. Also, only a certain range of frequency stimulations as reactivation destabilizes a consolidated LTP, which is regarded as a boundary condition for LTP reconsolidation.

## Methods

### Animals

All procedures involving the use of animals were conducted in compliance with the guidelines of the National Institutes of Health and were approved by the Animal Care and Use Committee of University of Toyama. Male Wistar ST rats 15 weeks of age were purchased from Japan SLC Inc. Food and water were provided ad libitum.

### Surgical procedure

Adult male Wistar rats (17–25 weeks old, >400 g) were used for implantation of cannula and electrodes. We used a surgical procedure described previously with a slight modification [8, 9]. Briefly, a bipolar stimulating electrode and a monopolar recording electrode made of tungsten wire were positioned stereotaxically to stimulate medial perforant pathways and its projections, while recording in the dentate gyrus. The stimulating electrode was positioned at 7.7–8.7 mm posterior, 4.2–5.3 mm lateral and 5.0–5.9 mm ventral to the bregma. The recording electrode was positioned ipsilateral at 4.0 m posterior, 2.5 mm lateral and 3.1–4.0 mm ventral to the bregma. For intraventricular (i.c.v.) infusion, a stainless-steel guide cannula (Eicom, Japan) was positioned ipsilateral at 0.8–1.0 mm posterior, 1.6 mm lateral, and 4.0 mm ventral to bregma. After surgery, a dummy cannula (Eicom), which extends 1.0 mm beyond the end of the guide cannula, was inserted into the guide cannula. Rats were allowed to recover for at least 10 days in individual home cages before experiment.

### Dentate gyrus LTP in unanaesthetized freely moving rats

LTP experiments on freely moving rats were performed as described previously with a slight modification [8–11]. After recovery from surgery, the intensity of the stimulus current to elicit the maximum population spike (PS) amplitude was determined for each animal. Three days later, LTP was induced by high frequency tetanic stimulation at 400 Hz, HFS400, consisting of 10 trains with 1 min intertrain intervals and each train consisted of 5 bursts of 10 pulses at 400 Hz, delivered at 1 s interburst intervals, a total of 500 pulses. The PS amplitude baseline response was monitored by delivering test pulses at 0.05 Hz (TP0.05) for 15 min before (base line) and 5 min after (post-LTP) HFS400. If potentiation is not induced immediately after HFS400, the rats were removed from data analysis. Three days after HFS400, reactivation was induced either by low frequency stimulations at 0.1 Hz (LFS0.1), theta stimulation at 8 Hz (TS8), or by high frequency stimulations at 400 Hz (HFS400). LFS0.1 consisted of 60 pulses at 0.1 Hz. TS8 consisted of 64 pulses at 8 Hz. HFS400 was induced as in the LTP induction. The PS amplitude baseline (TP0.05) was monitored for 15 min before (pre-reactivation) and 5 min after (post-reactivation) reactivation induction. In the no reactivation control group, no pre- or post-reactivation recordings were done to avoid any possibility for induction of reactivation. Immediately after the post-reactivation recording, rats received an i.c.v. drug infusion as described below. The PS amplitude baseline (TP0.05) was monitored for 15 min daily for three consecutive days after the reactivation session. The intensity of the stimulus current was set to elicit 40 % of the maximum PS amplitude for TP0.05, LFS0.1, and TS8, whereas 60 % of the maximum PS amplitude for HFS400.

### Drug infusion

Immediately after reactivation, dummy cannula was removed, and injection cannula (Eicom), which extends 0.5 mm beyond the end of the guide cannula, was inserted on unanesthetized rat. Drug was infused slowly via infusion pump at a rate of 1 μl/min into ventricle of freely moving rat. To inhibit protein synthesis, 600 μg/5 μl anisomycin was used. Anisomycin (Sigma) was dissolved in minimum HCl, diluted with PBS and adjusted to pH 7.4 with NaOH. To inhibit the N-methyl-D-aspartate receptors (NMDAR), 115.2 ng/4 μl CPP (3-((R)-2-Carboxypiperazin-4-yl)-propyl-1-phosphonic acid; Tocris) dissolved in saline was used. Following drug infusion, injection cannula was left in place for 5 min to allow drug diffusion.

### Immunohistochemistry

One hour after electroconvulsive stimulation (ECS), rats were deeply anesthetized with an overdose of pentobarbital solution, and removed brains were frozen in crushed dry ice and coronal sections of 14 μm thickness were cut on a cryostat and attached to a slide glass. Sections were fixed in 4 % paraformaldehyde and then incubated in PBS containing 4 % donkey serum (Chemicon, Temecula, CA) and 0.2 % Triton X-100 for 1 h at room temperature. Slices were incubated with rabbit c-fos antibody (1:500, sc-52, Santa Cruz Biotechnology, Inc, Santa Cruz, CA), and mouse anti-NeuN antibody (1:500, MAB377, Chemicon) overnight at 4 °C. After washing with PBS, the slices were incubated with Alexa Fluor 488-conjugated donkey anti-rabbit IgG (1:300, Molecular Probes, Eugene, OR), and Alexa Fluor 546-conjugated donkey anti-mouse IgG (1:300, Molecular Probes) for 2 h at room temperature. ProLong Gold Antifade Reagent (Molecular Probes) was used to protect samples from bleaching. Fluorescence images were obtained using the all-in-one fluorescence microscope, BZ-9000 (Keyence, Japan) through a 4× Plan-Apo objective (Nikon, Japan).

### Quantitative analysis of c-Fos in the dentate gyrus

Dentate gyrus was identified through the NeuN fluorescence (red channel), and region of interest (ROI) were manually positioned to fully cover the dentate gyrus. Average fluorescence intensities of c-Fos (green channel) in the ROI were calculated and the background intensities were subtracted. The fold changes relative to the average of the PBS group with ECS images were used for comparison.

### Statistics

Statistical analyses were performed using Prism 6.0. Two-way analysis of variance (ANOVA) with the indicated post-hoc test was used for factorial analysis. All data are reported as the mean ± standard error of mean (SEM).

## Abbreviations

CPP: (3-((R)-2-Carboxypiperazin-4-yl)-propyl-1-phosphonic acid
DG: dentate gyrus
ECS: electroconvulsive stimulation
E-LTP: early phase LTP
fEPSP: field excitatory postsynaptic potential
HFS: high frequency stimulation
i.c.v.: intracerebroventricular
LFS: low frequency stimulation
L-LTP: late phase LTP
LTD: long-term depression
LTM: long term memories
LTP: long term potentiation
NMDAR: N-methyl-D-aspartate receptor
PBS: phosphate buffered saline
PS: population spike
RM: repeated measures
STM: short-term memory
TS: theta stimulation
UPS: ubiquitin-proteasome system
